# RETRACTION: Inhibiting HMGB1 with Glycyrrhizic Acid Protects Brain Injury after DAI via Its Anti‐Inflammatory Effect

**DOI:** 10.1155/mi/9854643

**Published:** 2025-12-18

**Authors:** Mediators of Inflammation

RETRACTION: H. Pang, T. Huang, J. Song, D. Li, Y. Zhao, and X. Ma, “Inhibiting HMGB1 with Glycyrrhizic Acid Protects Brain Injury after DAI via Its Anti‐Inflammatory Effect,” *Mediators of Inflammation*, 2016, 4569521, https://doi.org/10.1155/2016/4569521.

The above article, published online on 06 March 2016 in Wiley Online Library (wileyonlinelibrary.com), has been retracted by agreement between the handling Editor, Dr. Vinod K. Mishra and John Wiley & Sons Ltd.

The retraction has been agreed due to errors identified in the figures. Initial concerns with Figure 2b were raised on PubPeer [1], where the Sham panel in Figure 2b is identical to the Sham panel in Figure 6 of [2]. Subsequent investigation also identified multiple inconsistencies with the blots in Figure 2c.

After assessment of the concerns and author’s response, the editorial board have deemed the results unreliable, and the article is therefore retracted.

Jinning Song disagrees with the retraction. No response was received from the remaining authors.
